# Prediction model for EBV infection following HLA haploidentical matched hematopoietic stem cell transplantation

**DOI:** 10.1186/s12967-024-05042-9

**Published:** 2024-03-06

**Authors:** Xun-Hong Cao, Ze-Ying Fan, Ying-Jun Chang, Lan-Ping Xu, Xiao-Hui Zhang, Xiao-Jun Huang, Xiang-Yu Zhao

**Affiliations:** Peking University People’s Hospital, National Clinical Research Center for Hematologic Disease, Peking University Institute of Hematology, Beijing Key Laboratory of Hematopoietic Stem Cell Transplantation, Beijing, China

**Keywords:** EBV, Allo-HSCT, The elders, Immune, Age

## Abstract

**Aims:**

Allogeneic hematopoietic stem cell transplantation (allo-HSCT) is an effective treatment for hematological malignancies. However, viral infections, particularly EBV infection, frequently occur following allo-HSCT and can result in multi-tissue and organ damage. Due to the lack of effective antiviral drugs, these infections can even progress to post-transplant lymphoproliferative disorders (PTLD), thereby impacting the prognosis. In light of this, our objective is to develop a prediction model for EBV infection following allo-HSCT.

**Methods:**

A total of 466 patients who underwent haploidentical hematopoietic stem cell transplantation (haplo-HSCT) between September 2019 and December 2020 were included in this study. The patients were divided into a development cohort and a validation cohort based on the timing of their transplantation. Our aim was to develop and validate a grading scale using these cohorts to predict the risk of EBV infection within the first year after haplo-HSCT. Additionally, single-cell RNA sequencing (sc-RNAseq) data from the bone marrow of healthy donors were utilized to assess the impact of age on immune cells and viral infection.

**Results:**

In the multivariate logistic regression model, four predictors were retained: donor age, female-to-male transplant, graft MNC (mononuclear cell) dose, and CD8 dose. Based on these predictors, an EBV reactivation predicting score system was constructed. The scoring system demonstrated good calibration in both the derivation and validation cohorts, as confirmed by the Hosmer–Lemeshow test (p > 0.05). The scoring system also exhibited favorable discriminative ability, as indicated by the C statistics of 0.72 in the derivation cohort and 0.60 in the validation cohort. Furthermore, the clinical efficacy of the scoring system was evaluated using Kaplan–Meier curves based on risk ratings. The results showed significant differences in EBV reactivation rates between different risk groups, with p-values less than 0.001 in both the derivation and validation cohorts, indicating robust clinical utility. The analysis of sc-RNAseq data from the bone marrow of healthy donors revealed that older age had a profound impact on the quantity and quality of immune subsets. Functional enrichment analysis highlighted that older age was associated with a higher risk of infection. Specifically, CD8 + T cells from older individuals showed enrichment in the pathway of “viral carcinogenesis”, while older CD14 + monocytes exhibited enrichment in the pathway of "regulation of viral entry into host cell." These findings suggest that older age may contribute to an increased susceptibility to viral infections, as evidenced by the altered immune profiles observed in the sc-RNAseq data.

**Conclusion:**

Overall, these results demonstrate the development and validation of an effective scoring system for predicting EBV reactivation after haplo-HSCT, and provide insights into the impact of age on immune subsets and viral infection susceptibility based on sc-RNAseq analysis of healthy donors' bone marrow.

**Supplementary Information:**

The online version contains supplementary material available at 10.1186/s12967-024-05042-9.

## Introduction

Allogeneic hematopoietic stem cell transplantation (allo-HSCT) is an effective treatment for malignant hematologic disorders and has gained popularity as a potential therapy for various malignant and non-malignant hematopoietic diseases. However, infection is a common and significant complication associated with post-transplantation mortality. Epstein-Barr virus (EBV) establishes a latent infection that persists throughout a person's life, but it can be reactivated in individuals with compromised immune systems, leading to disease development [1, 2]. Consequently, EBV reactivation is frequently observed following hematopoietic stem cell transplantation [3].

Reactivation of Epstein-Barr virus (EBV) can lead to uncontrolled proliferation of infected B cells, resulting in a condition known as post-transplant lymphoproliferative disease (EBV-PTLD) [4, 5]. Although the incidence of PTLD is relatively low, its high mortality significantly impacts patient prognosis [3, 6, 7]. Prophylaxis and preemptive therapy after transplantation have shown evidence of reducing EBV infection and its associated severe complications and poor outcomes [9, 10]. However, due to the complex transplantation environment and numerous factors influencing EBV reactivation, there is currently no clear predictive model for identifying individuals who would benefit from preemptive interventions. Several studies have attempted to identify independent risk factors for EBV viremia and the development of EBV-PTLD after allo-HSCT. These risk factors include T-cell depleted transplantation, more than two HLA mismatches, grade III to IV acute graft-versus-host disease (GVHD), and the use of the immunosuppressive agent ATG [3, 4, 6, 8]. However, a comprehensive predictive model for assessing the risk of EBV infection post-transplantation is lacking. Such a model would enable clinicians to effectively identify the high-risk population for EBV infection and implement appropriate intervention measures. Therefore, establishing a prediction model for EBV infection following transplantation is necessary and urgent.

Therefore, we have developed and validated the first simple scoring system capable of identifying post-transplant EBV infection. The dataset used for development and validation consisted of 466 patients who underwent allogeneic hematopoietic stem cell transplantation at our center, among which 95 patients developed EBV infection after transplantation. Additionally, we analyzed single-cell sequencing data from healthy donors and found that donor age is an important factor in the model. Our scoring system provides clinicians with a tool to stratify the risk of EBV occurrence in a timely and effective manner. By identifying high-risk populations, early prevention and intervention measures can be implemented. Ultimately, the implementation of this system will lead to improved patient outcomes and a reduced incidence of post-transplant EBV infection.

## Methods

### Study patients

**Fig. 1:**
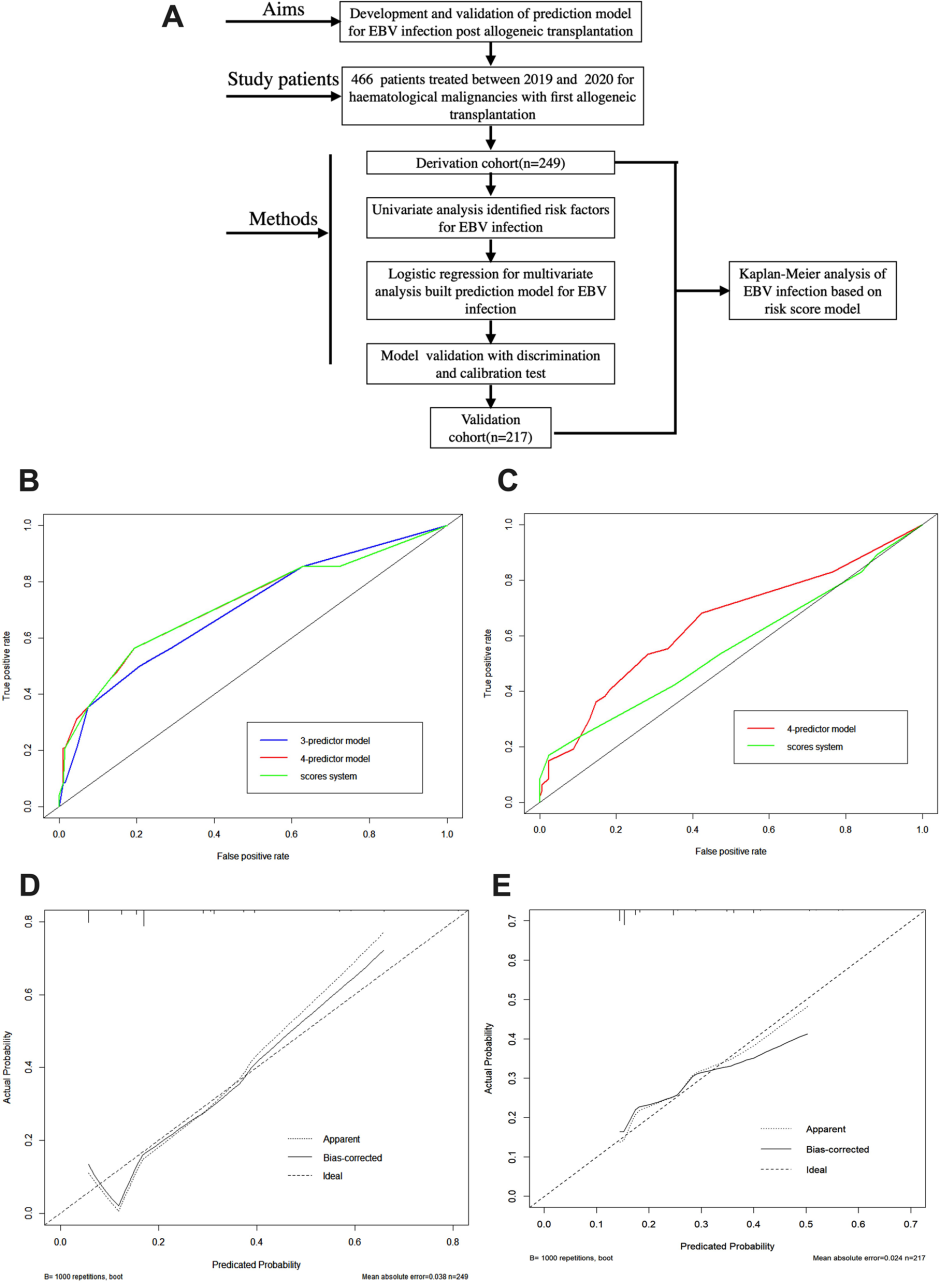
Discrimination capacity of the multivariate logistic regression model in the derivation and validation cohort. **A** Diagram of the study. **B** Receiver-operating characteristic curve of the 3-predictor model (donor age, female to male transplant and graft MNC dose), the final 4-predictor model (donor age, female to male transplant, graft MNC dose and graft CD8 dose), and the simplified score system in the derivation cohort. The C-statistics were 0.719 (95% CI 0.630–0.808; p < 0.001), 0.699 (95% CI 0.613–0.785; p < 0.001), and 0.718 (95% CI 0.630–0.807; p < 0.001), respectively. **C** Receiver-operating characteristic curve of the final 4-predictor model and simplified score system in the validation group. The C-statistic was 0.635 (95% CI 0.534–0.737; p = 0.006), and 0.601 (95% CI 0.499–0.703; p = 0.039) respectively. **D** Calibration plot in the derivation cohort (Hosmer–Lemeshow test, p = .702). **E** Calibration plot in the validation cohort (Hosmer–Lemeshow test, p = .261)

Between September 2019 and December 2020, a total of 466 patients underwent non-myeloablative conditioning, T-cell replete HLA haploidentical matched allo-HSCT (haplo-HSCT) from related donors at Peking University People’s Hospital. The patients had various diseases, including acute myeloid leukemia (AML), acute lymphoblastic leukemia (ALL), myeloid dysplasia syndrome (MDS), aplastic anemia (AA), chronic myeloid leukemia (CML), lymphoma, and more. We divided the patients into two groups based on the time of transplantation: one for the derivation cohort of the prediction model and the other for the validation cohort. To adapt to the updated transplantation protocol, we used the patient cohort from June 2020 to December 2020 as the derivation cohort, while the patient cohort transplanted from September 2019 to May 2020 was used as the validation cohort to verify the applicability of the model under different transplantation conditions. Of the 249 patients in the derivation cohort and 217 patients in the validation cohort, 48 and 47 patients, respectively, had EBV infection within 365 days post-HSCT. The study design was illustrated in Fig. 1A.Transplantation procedures were conducted according to previous studies [11, 12]. The study protocol was designed in accordance with the Declaration of Helsinki and was approved by the institutional review board of Peking University People’s Hospital.

### Definitions

Peripheral blood was collected regularly from all patients after transplantation, and EBV-DNA of plasma was detected by quantitative polymerase chain reaction (Q-PCR). EBV-DNA of peripheral blood was monitored 1–2 times per week from + 1 day to + 100 days after transplantation, once every 2 weeks after + 100 days, and once every 4 weeks from + 6 months to + 12 months. The frequency of testing was increased or decreased according to the patient’s condition.

EBV infection/reactivation was defined as > 500 copies/ml EBV-DNA in peripheral blood for at least once. The time from the transplantation date to the first positive Q-PCR result of EBV (> 500 copies/ml) was defined as the EBV reactivation time. The diagnosis of EBV-associated PTLD was defined as EBV viremia consistent with symptoms and/or signs and detection of EBV in tissue specimens [11].

### Procedures and statistics

We first included 17 variables in a univariate analysis based on clinical importance, scientific knowledge, and risk factors for EBV infection identified in previously published articles [7, 8]. Categorical variables were analyzed using the chi-square test and Fisher's exact test, while continuous variables were analyzed using the nonparametric Mann–Whitney test. We then used logistic regression for multivariate modeling, including variables with P < 0.15 in the univariate analysis. Polytomous variables were transformed into dichotomous variables based on the influence of each factor on the outcome, while continuous variables like donors or patients’ age, MNC count/kg or CD8 count/kg were transformed into dichotomous variables based on their maximum approximation index (sensitivity + specificity−1). Backward stepwise selection was used to identify variables for the multivariable logistic regression. Factors with P < 0.05 in the multivariate analysis were included in the final prediction model. The β coefficients derived from the final multivariate logistic regression were used to establish a scoring system for predicting EBV infection within 365 days post-HSCT [13, 14].

We evaluated the performance of our model by assessing its discrimination and calibration capabilities. Discrimination was evaluated by generating a receiver operating characteristic (ROC) curve and calculating a C-statistic. The C-statistic ranges from 0.5 to 1.0, with a higher value indicating better discrimination ability, i.e., better discrimination for patients with different outcomes. Calibration was evaluated using a calibration plot, which represents the relationship between the frequency of observed outcomes and the predicted probability, based on a bootstrapped sample of the study group. A well-calibrated model will have predictions that fit the 45-degree diagonal as closely as possible. Additionally, we used the Hosmer–Lemeshow test to evaluate the goodness-of-fit of the model. A P-value greater than 0.05 indicates that the model has extracted the information in the current data well and has a high goodness-of-fit. Using the predicted scores of the integral model, we divided patients into three risk groups (low, medium, and high) and plotted Kaplan–Meier curves to further evaluate the clinical efficacy of the model. We performed data analysis using SPSS 22.0 (IBM SPSS Statistics, IBM Corporation, Armonk, NY) and R studio 2.0 (RStudio, PBC, Boston, MA) [13, 14].

### Analysis of single-cell RNA data

The single-cell RNA-seq dataset used in this study was obtained from Karolyn's study [15]. All samples were regrouped according to age, with those over 50 years old classified as the elderly group and those less than or equal to 50 years old classified as the young group. Quality control metrics were performed based on Karolyn's study with minor modifications. Samples were analyzed using Seurat (https://satijalab.org/seurat/) with CCA and Louvain clustering, and visualized by UMAP. Differentially expressed genes (DEGs) were identified using the t-test between the young (≤ 50 years) and old (> 50 years) groups in each cell subset, with DEGs defined as those with an adjusted P-value < 0.05 and |Log2FC|> 0.25. To analyze the functional patterns of gene clusters and perform statistical analysis of DEGs, we used the Metascape web tool (www.metascape.org) for gene ontology and pathway enrichment analyses.

## Results

### Patient characteristics

In both the derivation and validation cohorts, the median time of EBV infection occurrence was 46 days (range, 25–212 days) and 47 days (range, 28–215 days) post haplo-HSCT, respectively. The cumulative incidence rate of EBV infection in the derivation cohort was 19.28% (48 out of 249 patients), while in the validation cohort, it was 21.66% (47 out of 217 patients). The demographic and clinical characteristics of patients in the two cohorts were similar, except for the graft source. In the derivation cohort, all patients received peripheral blood stem cells (PBSCs) as donor grafts in 2020 due to the impact of Covid-19. However, before 2020, most patients received a combination of bone marrow (BM) and PBSC allografts, so in the validation cohort, the graft source was primarily BM + PBSC. As a result of G-CSF mobilization, the percentage of CD34 + cells and lymphocytes were higher in peripheral blood (PB) compared to bone marrow (BM). Consequently, the cell counts of CD34 + cells and lymphocytes were higher in the derivation cohort than in the validation cohort. For a detailed overview of the demographic features and clinical characteristics of patients in the two cohorts, please refer to Table 1.

**Table 1:** Patient characteristics of the derivation and validation cohorts. The serological status of EBV was determined based on the IgM or IgG levels in patients or donors

	Derivation cohort (n = 249)	Validation cohort (n = 217)	P value
Patients age, median (range)	27 (1–66)	29 (1–64)	ns
Donors age, median (range)	39 (9–65)	36 (8–66)	ns
Patients gender, n(%)			ns
Male	152 (61.04)	135 (62.21)	
Female	97 (38.96)	82 (37.79)	
Donors gender, n(%)			ns
Male	185 (74.30)	156 (71.89)	
Female	64 (25.70)	61 (28.11)	
Donors age, n(%)			ns
> 50y	53(21.3%)	55(25.3%)	
≤ 50y	196(78.7%)	162(74.7%)	
ABO blood type, n(%)			ns
Match	127 (51.00)	123 (56.68)	
Minor mismatch	53 (21.29)	41 (18.89)	
Major mismatch	56 (22.49)	45 (20.74)	
Mismatch	13 (5.22)	8 (3.69)	
Donor-recipient relationship, n(%)			ns
Patrents to children	151 (60.64)	119 (54.84)	
Children to parents	56 (22.49)	56 (25.81)	
Sibling	42 (16.87)	42 (19.35)	
Donor-recipient gender, n(%)			ns
Male to male	114 (45.78)	98 (45.16)	
Male to female	71 (28.51)	59 (27.19)	
Female to female	25 (10.04)	21 (9.68)	
Female to male	39 (15.66)	39 (17.97)	
Stem cell source, n(%)			< 0.001
BM + PB	47 (18.88)	154 (70.97)	
PB	202 (81.12)	63 (29.03)	
Serological EBV status of patients and donors, n(%)			ns
P−D−	1 (0.40)	1 (0.46)	
P−D +	8 (3.21)	16 (7.37)	
P + D−	9 (3.61)	8 (3.69)	
P + D +	231 (92.77)	192 (88.48)	
HLA mismatch, n(%)			ns
1	15 (6.02)	14 (6.45)	
2	29 (11.65)	33 (15.21)	
3	205 (82.33)	170 (78.34)	
aGVHD, n(%)			ns
aGVHD-	150 (60.24)	101 (46.54)	
aGVHD grade 1–2	84 (33.74)	101 (46.54)	
aGVHD grade 3–4	15 (6.02)	15 (6.91)	
Count of grafts components/kg, median(range)			
MNC (10^8^/kg)	9.39 (6.30–20.25)	9.70 (5.48–22.45)	ns
CD34 (10^6^/kg)	3.95 (1.10–17.64)	2.78 (0.23–12.69)	< 0.001
Lym (10^6^/kg)	374.98 (91.28–960.46)	337.62(109.24–1057.09)	< 0.001
CD14 (10^6^/kg)	156.72 (55.11–348.05)	148.52 (51.71–305.82)	0.015
CD3 (10^6^/kg)	269.28 (64.62–650.48)	248.46 (61.95–645.75)	0.003
CD4 (10^6^/kg)	147.90 (38.39–414.48)	128.62 (35.68–358.13)	< 0.001
CD8 (10^6^/kg)	91.89 (21.00–277.85)	93.00 (20.64–297.16)	ns
NK (10^6^/kg)	37.47 (6.09–183.20)	29.12 (3.15–192.20)	0.005
EBV infection, n(%)	48 (19.28)	47 (21.66)	ns
Median time of EBV infection, days (range)	46 (25–212)	47 (28–215)	ns

### Establishment of the predictive model for EBV infection after allogeneic haplo-HSCT

**Fig. 2:**
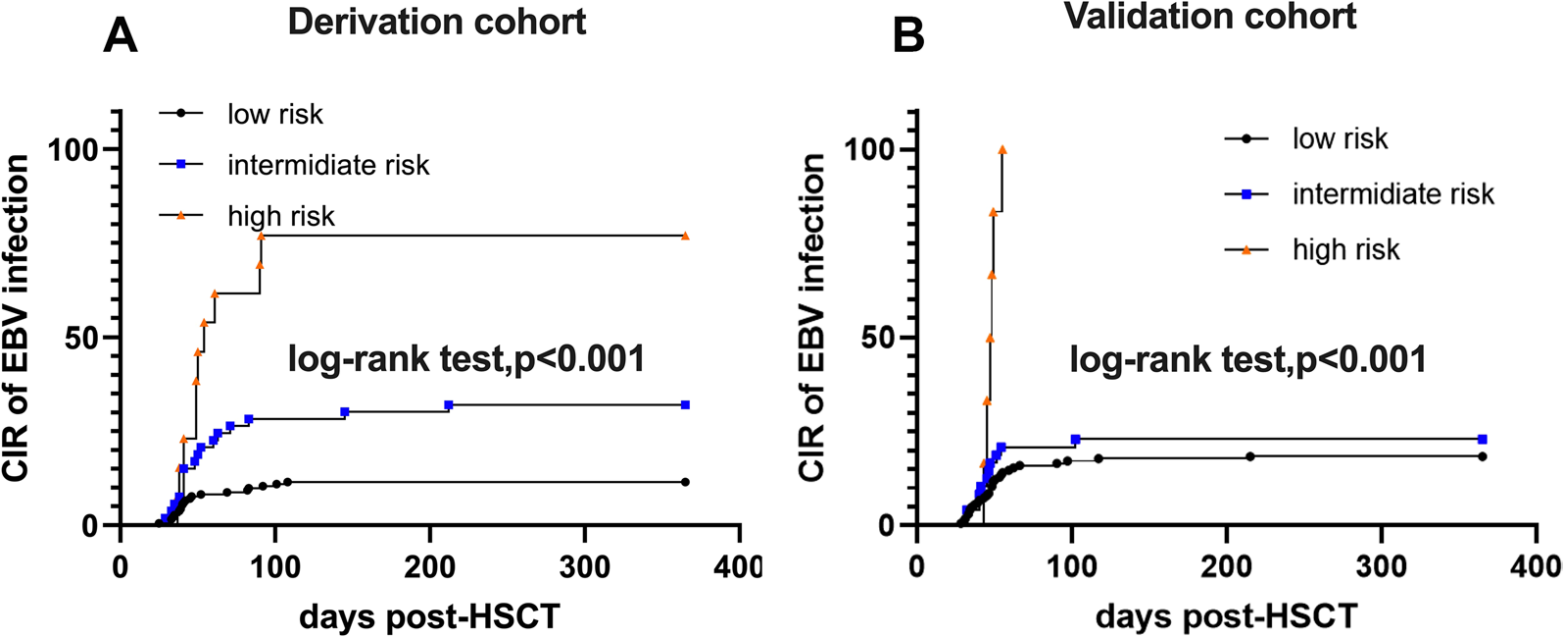
The cumulative incidence rate (CIR) of EBV infection in allo-HSCT patients according to the score system in the derivation and validation groups. High risk represents patients with scores of 8 to 11, intermediate risk represents patients with scores of 5 to 6, and low risk represents patients with scores of 0 to 3. **A** CIR of EBV infection in the derivation cohort (log-rank test, p < 0.001). **B** CIR of EBV infection in the validation cohort (log-rank test, p < 0.001)

In the study, Table 2 presents the results of the univariate analysis conducted to assess the variables that may affect EBV infection. A total of 17 variables were included in the derivation cohort analysis. Among these variables, six were found to be associated with EBV infection at a significance level of P < 0.15. These six variables were subsequently included in the multivariate analysis, as shown in Table 3. Using logistic regression analysis, three variables were identified as independent predictors of EBV infection post-haplo-HSCT at a significance level of P < 0.05. These predictors were older donor age, female to male transplant, and a high number of mononuclear cells (MNCs) in the grafts. Additionally, CD8 + T cell count in the graft was included in the final prediction model for several reasons. Firstly, previous studies have demonstrated a correlation between the number of CD8 + T cells in grafts and the rapid engraftment of donor T cells in patients post-HSCT [16, 17]. Secondly, although the P-value for CD8 + T cell count in the graft was 0.059, which is very close to the statistical significance threshold of 0.05, it was still considered relevant to include it in the model. In the final prediction model, consisting of four predictors, all four factors remained statistically significant (P < 0.05), as shown in Table 4. Above all, compared with the 3-factor prediction model (C-statistic: 0.699, 95% confidence interval [CI] 0.613–0.785), the model with CD8 + T cell count of grafts (C-statistic: 0.719, 95% CI 0.630–0.808) increased the discrimination ability (Fig. 1A). The final 4-predictor model had well goodness of fit according to the Hosmer–Lemeshow test (P = 0.263) and the calibration plot (Fig. 1C). The final 4 variables were incorporated in the nomograms to predict the probability of EBV infection after transplantation (Additional file 1: Fig. S1), but the nomograms are not simple enough in clinical application. Therefore, we incorporated these 4 variables into a simplified prediction score, each variable was weighted on the basis of its β coefficient obtained from the multivariable logistic regression. These predictors include older donor age, female to male transplant, high number of MNCs in grafts, and CD8 + T cell count in the graft. Accordingly, we separated patients with EBV infection post allo-HSCT into 3 risk groups: high risk (score, 8–11), intermediate risk (score, 5–6), and low risk (score, 0–3). The risk degree was able to distinguish the cumulative incidence rate of EBV infection post HSCT with different risks (P < 0.001) (Fig. 2A).

**Table 2:** Univariate analysis comparing patients with or without EBV infection within 365 days post-HSCT in the derivation cohort

	EBV−(n = 201)	EBV + (n = 48)	P value
Patients age, median(range)	26 (1–64)	29.5 (1–66)	0.339
Donors age, median(range)	37.5 (9–65)	42 (9–65)	**0.012**
Patients gender, n(%)			0.060
Male	117	35	
Female	84	13	
Donors gender, n(%)			0.178
Male	153	32	
Female	48	16	
ABO type, n(%)			**0.016**
Match	95	32	
Mismatch	106	16	
Donor-recipient relationship, n(%)			0.336
Patrents to children	118	33	
Children to parents	46	10	
Sibling	37	5	
Donor-recipient gender, n(%)			**0.004**
Female to male	25	14	
Others	176	34	
Stem cell source, n(%)			**0.106**
BM + PB	34	13	
PB	167	35	
Serological EBV status of patients and donors, n(%)			0.588
P−D−	1	0	
P−D +	8	0	
P + D−	8	1	
P + D +	184	47	
HLA mismatch, n(%)			0.405
1	14	1	
2	22	7	
3	165	40	
aGVHD, n(%)			0.331
aGVHD−	123	27	
aGVHD grade 1–2	68	16	
aGVHD grade 3–4	10	5	
Count of grafts components/kg, median(range)			
MNC (10^8^/kg)	9.29 (5.01–20.25)	10.27 (5.3–19.15)	**0.028**
CD34 (10^6^/kg)	3.42 (0.67–17.64)	3.39 (1.28–13.09)	0.575
Lym (10^6^/kg)	370.30 (91.28–960.46)	385.54 (151.18–640.10)	0.666
CD14 (10^6^/kg)	157.82 (55.11–348.05)	155.32 (84.31–360.26)	0.369
CD3 (10^6^/kg)	255.03 (64.62–650.48)	254.01 (74.32–496.17)	0.585
CD4 (10^6^/kg)	139.65 (38.39–414.48)	141.61 (43.69–331.41)	0.846
CD8 (10^6^/kg)	89.05 (20.17–277.85)	84.95 (24.87–206.48)	**0.138**
NK (10^6^/kg)	35.96 (6.09–183.20)	30.28 (7.27–107.07)	0.347

**Table 3:** Odds ratios for categorical and dichotomized continuous variables with a P < 0.15 in univariate analysis

	Dichotomized value	Odds ratio (95%CI)	p
Donor's age	≥ 50 y	2.513 (1.257–5.025)	0.009
ABO type	Match	2.232 (1.152–4.322)	0.017
Donor-recipient gender	Female to male	0.899 (1.369–6.138)	0.005
Stem cell source	BM + PB	1.824 (0.874–3.807)	0.109
MNC count/kg	≥ 9.8	2.646 (1.375–5.095)	0.004
CD8 count/kg	< 62.0	2.141 (1.092–4.197)	0.027

**Table 4:** Results of the multivariable logistic regression model for the derivation cohort

Characteristics	β coefficient	P	OR (95%CI)	Assigned scores
Donor’s age ≥ 50	1.094	0.006	2.986(1.378–6.473)	3
Female to male	1.179	0.005	3.252(1.431–7.392)	3
MNC count/kg ≥ 9.8	1.191	0.001	3.292(1.620–6.687)	3
CD8 count/kg < 62.0	0.794	0.034	2.213(1.063–4.606)	2

### Validation of the predictive model for EBV infection

When applying the 4-predictor model and score system to the validation cohort, we observed that the 4-predictor model exhibited good discrimination ability, as indicated by a C-statistic of 0.635 (95% CI 0.534–0.737) with a statistically significant p-value of 0.006. On the other hand, the score system demonstrated minor discrimination ability, with a C-statistic of 0.601 (95% CI 0.499–0.703). However, the score system still showed capability in distinguishing patients with and without EBV infection, as evidenced by a statistically significant p-value of 0.039 (Fig. 1B). The model was then fully calibrated in the validation cohort, as demonstrated by the Hosmer–Lemeshow test with a p-value of 0.296 and the calibration plot (Fig. 1D). This indicates that the predicted probabilities from the model align well with the observed probabilities of EBV infection in the validation cohort. Furthermore, the cumulative incidence of EBV infection post-haplo-HSCT was higher in the high-risk group identified by the score system (Table 5, Fig. 2B). This confirms that the score system effectively stratifies patients into different risk groups based on their likelihood of developing EBV infection.

**Table 5:** The cumulative incidence of EBV infection in patients post-HSCT by risk group

Risk group	score	Derivation cohort	Validation cohort	All patients
low	0–3	21/183 (11.5%)	30/163 (18.4%)	51/347 (14.7%)
intermediate	5–6	17/53 (32.1%)	11/48 (22.9%)	28/101 (27.7%)
high	8–11	10/13 (76.9%)	6/6 (100%)	16/19 (84.2%)

### The distribution and function of immune cells were affected by age

**Fig. 3:**
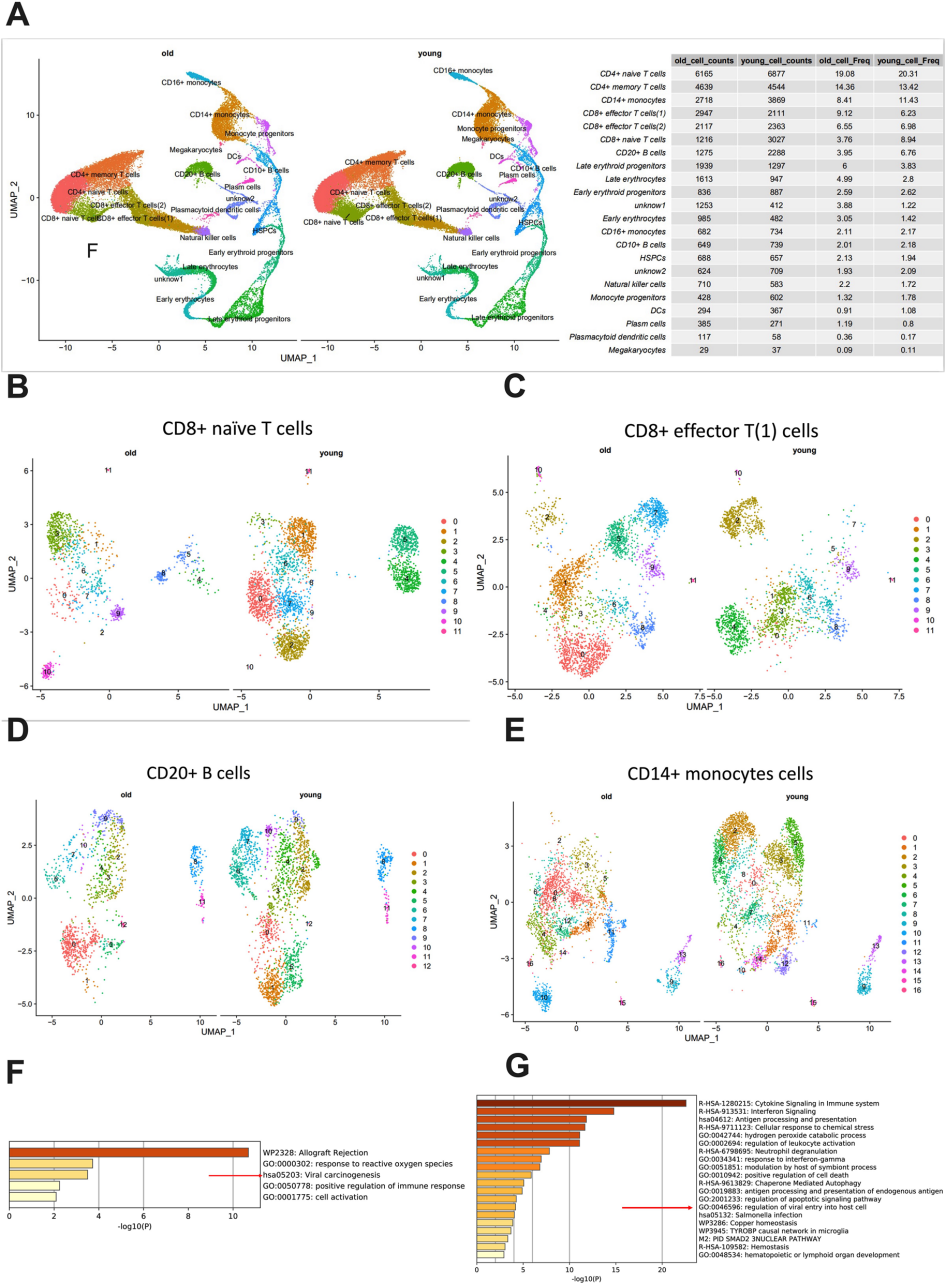
Age affects distribution of immune cell subsets. **A** Landscape of immune cell distribution between the older(> 50y) and the younger(≤ 50y). **B** Subsets analysis of CD8 naïve T cells between the two age groups. **C** Subsets analysis of CD8 + effector T (1) cells between the two age groups. **D** Subsets analysis of CD20 + B cells between the two age groups. **E** Subsets analysis of CD14 + monocytes cells between the two age groups. **F** GO pathways of upregulation genes in the older group of CD20 + B cells. **G** GO pathways of upregulation genes in the older group of CD14 + monocytes cells

In order to investigate whether donor age mediates the occurrence of EBV infection post-transplantation by influencing immune cell status, we analyzed single-cell RNA sequencing data from the bone marrow of healthy donors in Karolyn’s study [15]. The analysis revealed significant differences in various immune cell populations between younger and older donors, with the age threshold set at 50 years. Specifically, older donors exhibited higher levels of CD8 + effector T cells and different stages of erythroid cell differentiation. On the other hand, younger donors had higher levels of CD8 + naïve T cells, CD14 + monocytes, and CD20 + B cells. However, there was no significant difference in the proportion of CD4 + naïve T cells and CD4 + memory T cells between the two groups. These findings suggest that age may play a role in the regulation of immune cell populations (Fig. 3A). To further explore the functional differences within these immune cell subsets between the two age groups, we conducted a comparison of subpopulations within CD8 + naïve T cells, CD8 + effector T cells, CD20 + B cells, and CD14 + monocytes. Notably, each immune subset exhibited a more refined classification of subpopulations, and significant differences were observed (Fig. 3B–E), indicating that donor age may influence the immune cell composition, potentially contributing to the regulation of immune responses and thereby impacting the occurrence of EBV infection after transplantation.

The differential expression gene (DEG) analysis and Gene Ontology (GO) analysis of CD8 + naïve T cells, CD8 + effector T cells, and CD14 + monocytes between older and younger donors revealed interesting findings. In CD20 + B cells of older donors, there was enrichment of pathways related to viral carcinogenesis and allograft rejection. This enrichment was driven by the upregulation of genes such as HLA-B, HLA-C, H4C3, IGHA1, NKG7, GNLY, and CD79B (Fig. 3F). These findings suggest that the CD20 + B cells of older donors may exhibit a gene expression profile that is associated with increased susceptibility to viral infections and higher risk of allograft rejection. Additionally, in CD14 + monocytes of older donors, the GO analysis revealed enrichment of the pathway "regulation of viral entry into host cells" (Fig. 3G). This suggests that CD14 + monocytes from older donors may have altered regulatory mechanisms related to viral entry into host cells, potentially influencing their ability to respond to viral infections. These results emphasize the importance of considering donor age in the selection of donors for transplant recipients, particularly in relation to the risk of EBV infection post-transplantation.

## Discussion

In this study, we have identified several risk factors for EBV infection following HSCT (hematopoietic stem cell transplantation). These factors include donor age in the elderly, a female donor matched with a male recipient, high doses of MNCs (mononuclear cells), and low doses of CD8 + T cells. Based on these factors, we have developed a predictive model for EBV infection that demonstrates good clinical efficacy and can accurately distinguish patients at a high risk of infection. Furthermore, our analysis of single-cell sequencing data has revealed that donor age significantly impacts the quantity and quality of immune cells. This finding highlights the importance of considering donor age in the selection of donors for transplant recipients.

Our study highlights the relationship between donor age and EBV infection post-HSCT. Previous studies have indicated that older donors are associated with higher non-relapse mortality (NRM) and poorer survival outcomes after transplantation18. The exact mechanisms underlying these associations are not yet well understood but may be attributed to age-related declines in hematopoietic function and immunity [19–21]. In the elderly, hematopoietic stem cells (HSCs) exhibit signs of senescence, characterized by changes in transcriptional and epigenetic profiles, increased levels of reactive oxygen species (ROS), and DNA damage, ultimately leading to HSC senescence. Furthermore, HSCs from older donors demonstrate reduced migration to the bone marrow and regenerative capacity, and they have a tendency to differentiate into myeloid cells, resulting in an increased production of myeloid progenitor cells and a decreased production of lymphoid progenitor cells [19, 20]. Consistent with HSC senescence, patients who receive transplants from older donors often experience inadequate and imbalanced immune reconstitution [22–24].

To investigate the direct effects of age on donor immune cells, we analyzed the subsets and functions of immune cells from younger and older healthy donors using a database. Our findings revealed that not only CD8 + effector T cells increased in older donors but also CD8 + naïve T cells, CD14 + monocytes, and CD20 + B cells were decreased, with significant differences observed in the classification of these subsets between younger and older donors. Gene pathway enrichment analysis also identified an enrichment of virus infection and pathogenic genes in CD20 + B cells and CD14 + mononuclear cells from older donors. These findings align with the characteristics of immunosenescence, characterized by decreased proliferation and function [21, 25–27], and they may explain the impact of donor age on the incidence of EBV infection following transplantation. Our study provides insights into the underlying mechanisms of age-related changes in the immune system and holds important implications for the development of personalized therapies and interventions aimed at enhancing immune function and preventing age-related diseases.

Our study has revealed a strong association between female-to-male grafts and EBV infection following HSCT. This finding aligns with a previous small cohort study involving 102 patients who underwent allo-HSCT, which also identified female donors as a risk factor for EBV reactivation after transplantation [28]. Interestingly, another study reported that transplantation with a female donor to a male recipient was associated with an increased risk of invasive mold infections after allo-HSCT, although the underlying mechanisms for this relationship remain unclear [29]. Additionally, Wang et al. reported that female donors, particularly when the recipient was male, experienced higher non-relapse mortality and worse survival outcomes after transplantation [18]. The mechanisms underlying the influence of female donors on EBV infection and poor outcomes post-transplantation require further investigation. It is possible that gender-related differences in immune response and susceptibility to viral infections contribute to these observations, although other factors such as variations in HLA-matching or other genetic factors may also play a role. Further research is necessary to gain a better understanding of these mechanisms and to develop strategies aimed at reducing the risk of EBV infection and other complications following HSCT.

Our study also revealed a significant association between the dose of CD8 + T cells in the graft and EBV infection following HSCT. This finding is consistent with previous research indicating that the CD8 + T cell dose in the graft correlates with transplantation outcomes in reduced-intensity conditioning allo-HSCT. Studies have demonstrated that a higher graft CD8 + T cell dose predicts better transplant outcomes, improved progression-free survival and overall survival, as well as a reduced risk of primary disease relapse. These positive outcomes are primarily attributed to the accelerated engraftment of donor T cells in patients with higher CD8 + T cell doses [16, 17]. Therefore, it is plausible that a high CD8 + T cell dose in the graft predicts rapid T cell reconstitution post-HSCT, which could potentially provide protection against EBV reactivation [36, 37]. These findings suggest that optimizing the composition and dose of immune cells in the graft may represent an important strategy to mitigate the risk of EBV infection and enhance outcomes following HSCT. Further research is necessary to explore the precise mechanisms underlying the association between CD8 + T cell dose and EBV infection after HSCT.

Interestingly, our study revealed that a high number of grafts MNCs (mononuclear cells) was identified as an independent risk factor for EBV infection following transplantation. It is widely reported and clinically recognized that a high MNC dose, along with CD34 cell dose, promotes the engraftment of neutrophils and platelets post-transplant. However, there have been no previous studies reporting that a high MNC dose could have adverse effects on transplantation outcomes. This highlights the need for further investigation into the impact of MNC dose on complications and outcomes after transplantation. Additional research in this area would help provide a better understanding of the relationship between MNC dose and the risk of EBV infection, as well as other potential complications following transplantation.

Previous studies have identified several risk factors for EBV viremia and EBV-PTLD post-HSCT, including T cell-depleted transplantation, the use of ATG, HLA mismatch of more than 2 loci, severe acute GVHD, elderly transplant recipients, umbilical cord blood transplantation, and donor-recipient EBV serological status before transplantation, especially donor serologically positive and recipient serologically negative [6–8]. In our study, all patients received in vivo T-cell depletion allogeneic haplo-HSCT with ATG in the “Beijing protocol” mode. Since the number of cells from umbilical cord blood is insufficient for transplantation, our cohort did not include any patients who received umbilical cord blood transplantation alone. There were no statistically significant differences in the number of HLA-mismatched loci, severe acute GVHD, or donor-recipient EBV serological status between patients with and without EBV infection in our cohort. We did not find patient age to be a risk factor for EBV infection, possibly due to cohort heterogeneity between different studies.

One of the strengths of our study is the relative consistency of the transplant patients, as all of them underwent allogeneic haploidentical HSCT. However, due to the size of our cohort, we were unable to analyze certain potentially important variables, including EBV serological status, HLA mismatched loci, and severe acute GVHD. Therefore, the score system model we developed requires validation in larger cohorts and external studies. Additionally, our results did not allow for the development of a prediction model specifically for EBV-related disease or PTLD, indicating the need for further investigation in this area.

In summary, our study identified several independent risk factors for EBV infection post-HSCT, including donor age, female-to-male transplant, infused graft MNC, and CD8 cell dose. Additionally, the analysis of single-cell sequencing data revealed significant alterations in the distribution of cell subsets and cellular pathways of immune cells related to donor age. We successfully developed a straightforward and practical score system model using multivariable regression, which enables the prediction of EBV infection risk following transplantation. This model incorporates easily accessible clinical factors, enhancing the applicability and accessibility of the predictive tool.

### Supplementary Information

**Additional file 1: Figure S1.** Nomogram to predict the probability of EBV infection after transplantation.

## Data Availability

The raw data supporting the conclusions of this article will be made available by the authors, without undue reservation.
