# Supplementary figures and images for: Prediction model for EBV infection following HLA haploidentical matched hematopoietic stem cell transplantation
Source: J Transl Med. 2024 Mar 6;22:244. doi: 10.1186/s12967-024-05042-9 (PMC10916301; doi:10.1186/s12967-024-05042-9)

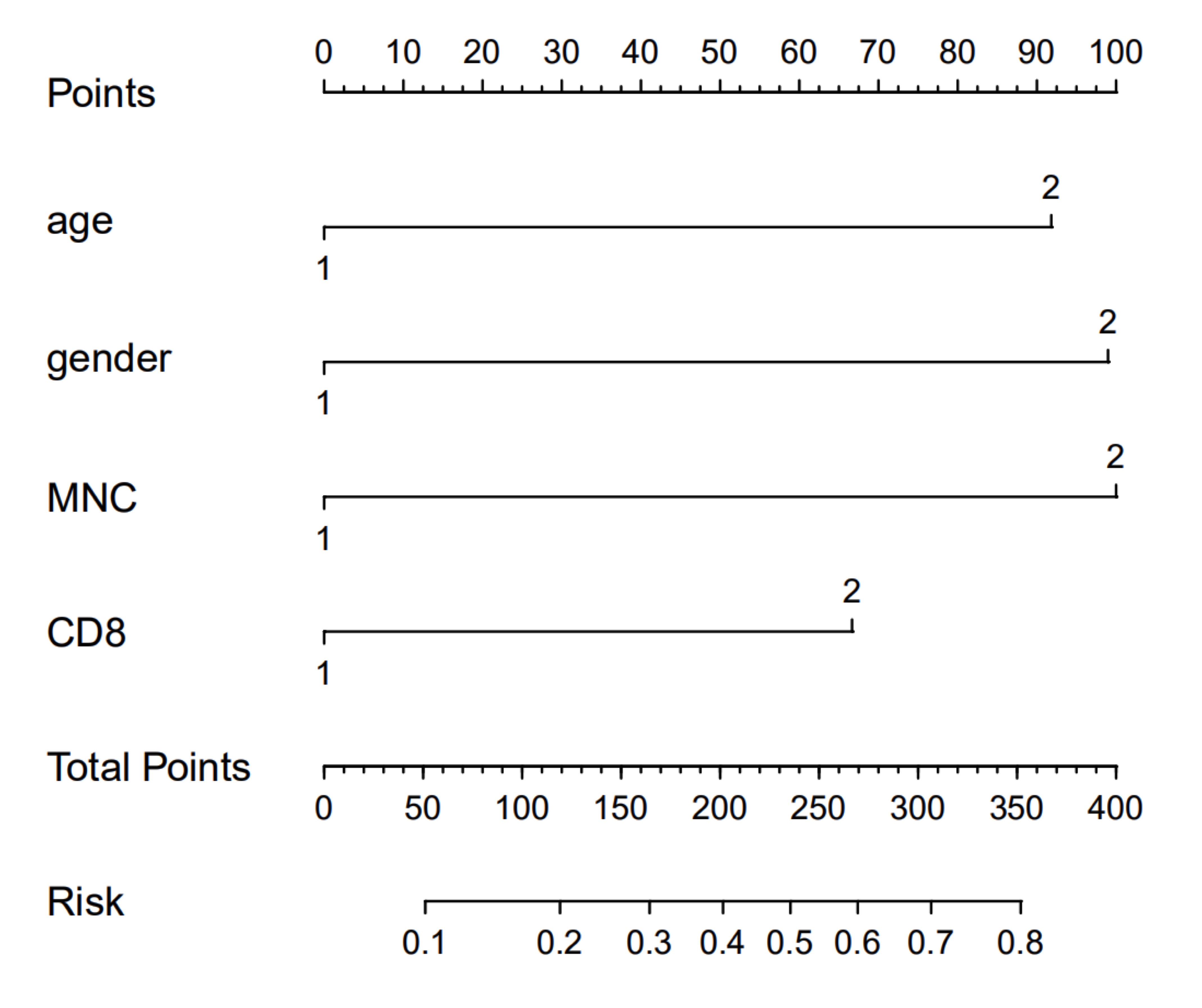

Supplement: Supplementary file 1 — Additional file 1: Figure S1. Nomogram to predict the probability of EBV infection after transplantation. [file 12967_2024_5042_MOESM1_ESM.jpg]
